# Fundamental Limitation in Electrochemical Methane Oxidation to Alcohol: A Review and Theoretical Perspective on Overcoming It

**DOI:** 10.1002/advs.202301912

**Published:** 2023-09-22

**Authors:** M.R. Ashwin Kishore, Sungwoo Lee, Jong Suk Yoo

**Affiliations:** ^1^ Department of Chemical Engineering University of Seoul Seoul 02504 Republic of Korea

**Keywords:** carbonate ions, density functional theory, electrochemical methane oxidation, limitation, mechanistic insights, oxidants

## Abstract

The direct conversion of gaseous methane to energy‐dense liquid derivatives such as methanol and ethanol is of profound importance for the more efficient utilization of natural gas. However, the thermo‐catalytic partial oxidation of this simple alkane has been a significant challenge due to the high C−H bond energy. Exploiting electrocatalysis for methane activation via active oxygen species generated on the catalyst surface through electrochemical water oxidation is generally considered as economically viable and environmentally benign compared to energy‐intensive thermo‐catalysis. Despite recent progress in electrochemical methane oxidation to alcohol, the competing oxygen evolution reaction (OER) still impedes achieving high faradaic efficiency and product selectivity. In this review, an overview of current progress in electrochemical methane oxidation, focusing on mechanistic insights on methane activation, catalyst design principles based on descriptors, and the effect of reaction conditions on catalytic performance are provided. Mechanistic requirements for high methanol selectivity, and limitations of using water as the oxidant are discussed, and present the perspective on how to overcome these limitations by employing carbonate ions as the oxidant.

## Graphical abstract

Electrochemical conversion of methane to energy‐dense liquid derivatives like methanol and ethanol is crucial for maximizing the use of natural gas. This work focuses on recent advancements in methane oxidation to alcohols. Mechanistic requirements for high methanol selectivity and limitations of using water as the oxidant are discussed. A theoretical perspective of utilizing carbonate ions is presented to overcome these limitations.

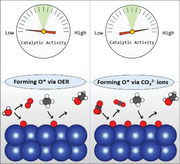

## 1 Introduction

**Figure 1:**
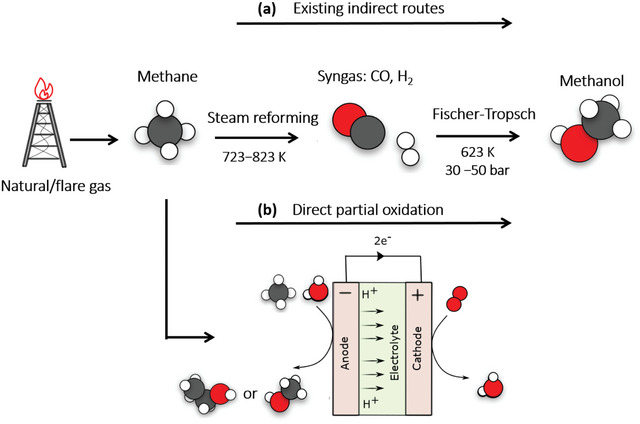
Schematic diagram of methane oxidation a) via existing indirect routes through steam reforming followed by Fischer–Tropsch synthesis. b) Direct partial oxidation using the electrocatalysis process.

Natural gas, primarily composed of methane, has played a vital role in meeting global energy demand due to its abundance and environmental benefits over conventional fossil fuels.^[^ ^1^, ^2^ ^]^ However, the storage and transportation of methane gas, particularly when it is produced as a byproduct at oil production sites, have been challenging, resulting in gas flaring in oil fields worldwide. In 2021, global gas flaring exceeded 143 billion cubic meters, equivalent to the total volume of natural gas imported into South Korea and Japan combined in the same year.^[^ ^3^ ^]^ This flaring practice results in the release of five times more methane into the atmosphere than previously estimated by researchers.^[^ ^4^ ^]^ The development of remote‐deployable technology capable of converting methane gas to easily transportable liquid derivatives, such as alcohol, could pave the way for more efficient utilization of natural gas. However, most commercially viable methane‐to‐alcohol conversion processes currently involve an indirect route via synthetic gas, which is energy‐intensive and expensive (**Figure** **1**a).^[^ ^5^ ^]^

An attractive alternative is to develop a catalytic process that can directly partially oxidize methane to alcohol under mild conditions, enabling small‐scale and decentralized productions in remote locations such as oil fields (Figure 1b).^[^ ^6^ ^]^ However, catalytic partial oxidation of methane to methanol under mild conditions has long been considered a “holy grail” reaction in catalysis due to the challenges of activating methane. Methane has a symmetrical tetrahedral structure with a high C−H bond dissociation energy of 438.8 kJ mol^−1^.^[^ ^7^ ^]^ Thus, strong reactants and harsh reaction conditions (high‐temperature, high‐pressure, and expensive operation processes) are required, but these conditions thermodynamically promote complete oxidation instead of partial oxidation.^[^ ^8^ ^]^

On the other hand, methane monooxygenases (MMOs) in methanotrophic bacteria have been successful in partially oxidizing methane to methanol under ambient conditions. Inspired by the iron‐oxo active sites in MMOs,^[^ ^9^, ^10^ ^]^ significant efforts have been made to develop thermal catalysts that can directly convert methane to methanol.^[^ ^11^, ^12^, ^13^, ^14^, ^15^ ^]^ For example, iron‐ and copper‐exchanged zeolites have been developed, showing promising results with the methane‐to‐methanol turnover frequency (TOF) of 23.9 mol CH_3_OH molcat^−1^ h^−1^ using O_2_ as the oxidant, and 2278 mol CH_3_OH molcat^−1^ h^−1^ using the relatively expensive H_2_O_2_ as the oxidant.^[^ ^16^, ^17^, ^18^, ^19^, ^20^ ^]^ However, these catalysts are all based on a temperature‐varying multi‐step process in which; 1) oxygen of the oxo‐metallic site is used to oxidize methane to methanol at low temperature (≈150 °C); 2) the oxidant fills in the oxygen vacancy to regenerate the oxo‐metallic site at high temperature (≈450 °C); and 3) the water solvent is injected to extract methanol from the catalyst surface at low temperature (≈150 °C).^[^ ^21^ ^]^ Recently, Roma−n‐Leshkov et al. have explored the possibility of using copper‐exchanged zeolites to continuously produce methanol using methane, oxygen, and water simultaneously at a constant temperature of 200 °C, but the conversion rate was found to be extremely low (0.001%).^[^ ^22^ ^]^

One fundamental reason for the failure of directly converting methane to alcohol using thermal catalysis is that the resulting product, alcohol, is more reactive than methane. Compared to methanol, methane activation via the radical pathway is difficult due to its higher homolytic bond dissociation energy, while the activation via the surface‐stabilized pathway is complex due to the symmetric structure and weakly polarized C−H bonds of methane.^[^ ^23^ ^]^ To address this issue, researchers need to find ways to stabilize alcohol relative to methane. One effective approach could be to utilize an aqueous environment, which allows the alcohol to be stabilized through solvation.

Utilizing electrocatalysis to convert methane into alcohol can serve as an appealing alternative method, given that water is employed as an oxidizing agent to partially oxidize methane. The method presents several advantages, such as maintaining room temperature throughout the process, continuous regeneration of oxo‐metallic sites by forming oxygen species from the water solvent under operating conditions, and the presence of water to facilitate the extraction of alcohol from the catalyst surface via solvation.^[^ ^24^, ^25^, ^26^ ^]^ However, the electrocatalytic conversion of methane to alcohol is restricted by a fundamental tradeoff between catalytic activity and selectivity, as discussed below.

For a methane conversion catalyst to be highly active, it must bind surface oxygen species, such as O* and OH*, extremely weakly to effectively activate methane via the O‐/OH‐assisted mechanism.^[^ ^27^, ^28^ ^]^ However, the consequence is that a relatively high potential must be applied to form OH* and/or O* from water. At this high potential, the oxygen species are vulnerable to complete oxidation via the oxygen evolution reaction (OER), which significantly decreases the faradaic efficiency of methane conversion.^[^ ^29^ ^]^ Iron oxides and iridium oxides are examples of catalyst materials that may encounter this fundamental limitation. Although these catalysts exhibit excellent performance for thermocatalytic methane oxidation due to their weak oxygen binding characteristics, they have seldom been employed for electrocatalytic methane oxidation due to their high OER activities.

Numerous reviews have been published on the partial oxidation of methane to oxygenates, covering a broad range of topics, such as different classes of thermo‐catalyst materials, including transition metals,^[^ ^30^, ^31^, ^32^, ^33^ ^]^ oxides,^[^ ^34^ ^]^ and metal‐organic frameworks.^[^ ^35^ ^]^ Others focus on electrocatalytic systems, highlighting different electro‐catalyst materials,^[^ ^36^, ^37^ ^]^ electrolytes,^[^ ^38^ ^]^ reaction conditions such as pressure, temperature, pH, and reactor design,^[^ ^26^ ^]^ mechanistic understanding of C−H bond activation,^[^ ^39^ ^]^ reaction pathways leading to different products,^[^ ^38^, ^40^ ^]^ and theoretical modeling focusing on the activity trends.^[^ ^41^ ^]^ Despite these efforts, there has been a lack of comprehensive analysis of the fundamental limitation of current electro‐catalytic systems in aqueous environments and how to overcome them. In this review, we provide an overview of the current progress in electrochemical methane oxidation, including mechanistic insights into methane activation and catalyst design principles based on descriptors, challenges, and potential solutions in electrocatalytic methane oxidation, the role of employing different oxidants, and effect of reaction conditions on catalytic performance, such as electrolyte, pressure, and temperature. Next, we present our perspective on the limitation of current electro‐catalytic methane‐to‐oxygenates conversion systems and suggest using CO_3_^2−^ ions as outsourced oxidants instead of conventional water oxidants. Although this is not our discovery, we originally explain why this approach is far more appealing. Finally, we highlight future research directions to produce alcohol from partial methane oxidation, which would be a valuable contribution to this field.

## 2 Current Progress on Electrochemical Methane Oxidation

### 2.1 Mechanistic Insights on Methane Activation and Catalyst Design Principles Based on Descriptors

**Figure 2:**
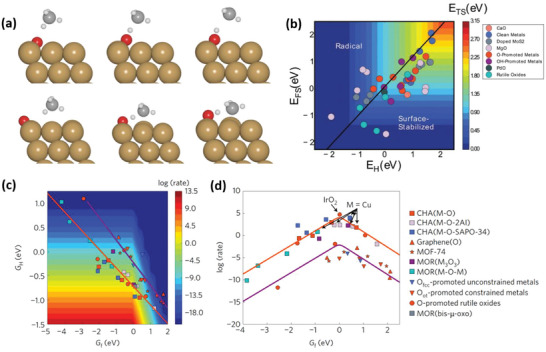
a) Structural representations of the initial state (left) transition state (middle), and final state (right) for the radical (top panel) and surface‐stabilized (bottom panel) methane activation pathways on a catalyst surface. b) A 2D volcano plot showing *E*_TS_ (transition energy) as functions of *E*_H_ (hydrogen adsorption energy) and E_FS_ (final state energy) as descriptors for the radical and surface‐stabilized methane activation pathways, respectively. The black line on the plot is described by the Equation, *E*_FS_  = 1.12*E*_H_ + 0.07, and indicates where the two descriptors yield equal values of *E*_TS_. Reproduced with Permission.^[^ ^28^ ^]^ Copyright 2017, Royal Society of Chemistry. c) A 2D volcano plot for the intrinsic rate of methane activation using *G*_H_ (hydrogen adsorption free energy) and *G*_f_ (active oxygen‐site formation free energy) as descriptors. d) A 1D volcano plot for the intrinsic rate of methane activation using *G*_f_ as a descriptor. Reproduced with permission.^[^ ^42^ ^]^ Copyright 2017, Springer Nature.

The process of methane oxidation begins by breaking one of the methane's four C−H bonds, known as the methane activation step. This C−H bond dissociation may occur through either the radical or surface‐stabilized pathway (as shown in **Figure** **2**a).^[^ ^34^ ^]^ In the radical pathway, atomic oxygen on the catalyst surface removes an electrophilic hydrogen atom (∙H) from methane, leading to the formation of a radical‐like ∙CH_3_ and OH∙.^[^ ^42^ ^]^ The resulting ∙CH_3_ interacts weakly with the catalyst surface through OH* without forming a chemical bond. On the other hand, the surface‐stabilized pathway starts with the heterolytic fission of the C−H bond, leading to the formation of CH_3_^−^ and H^+^.^[^ ^23^, ^29^, ^43^ ^]^ The resulting CH_3_^−^ coordinates strongly with the catalyst surface to form CH_3_*, while H^+^ is accepted by either surface atomic metal (M) or oxygen to form H*. Depending on the nature of the catalyst surface, the M−CH_3_* σ‐bond can be generated by σ‐bond metathesis, oxidative addition, or electrophilic activation.^[^ ^14^, ^23^, ^43^ ^]^

The descriptors‐based approach^[^ ^44^, ^45^ ^]^ that employs linear scaling relations^[^ ^46^ ^]^ to quickly predict the transition‐state energies of catalytic reactions is widely accepted as an effective means of screening and designing catalyst materials, obviating the need for resource‐intensive barrier calculations and extensive catalytic testing. However, since methane activation can occur via one of the two possible pathways that lead to distinctly different transition‐state scaling behaviors, two different descriptors have been proposed. For the methane activation systems that proceed through the radical pathway, the transition‐state energy (E_TS_) scales with the hydrogen affinity (E_H_) of the active site (as shown in the top panel of Figure 2a), while, for those that proceed through the surface‐stabilized pathway, it scales with the final‐state energy (E_FS_), which is determined by the binding energy of both H* and CH_3_* to the surface (as shown in the bottom panel of Figure 2b).

To accurately determine the transition energies as well as intrinsic rates for methane activation, it is crucial to determine the relevant methane activation pathway. Nørskov et al. compared the E_TS_’s for the radical and surface‐stabilized pathways for various classes of catalyst materials and found that the descriptor‐based approach does not always offer a straightforward means to determine the relevant pathway for most catalyst materials.^[^ ^28^ ^]^ For example, the black line in Figure 2b indicates where the two different methane activation pathways lead to equal values of *E*_TS_. Materials located above the black line (*E*_FS_ > 1.12*E*_H_ + 0.07, i.e., materials with high *E*_FS_ and low *E*_H_) follow the radical pathway, whereas those located below the black line (*E*_FS_ < 1.12*E*_H_ + 0.07, i.e., materials with high *E*_H_ and low *E*_FS_) follow the surface‐stabilized pathway. Interestingly, many catalyst materials, particularly those with higher values of descriptors, tend to cluster along the black line, indicating that both methane activation pathways are comparably feasible and that the preferred pathway relies more on the catalyst geometry. For example, the surface‐stabilized route may be hindered by the spatial separation of the active sites, regardless of CH_3_* stability. Conversely, the surface‐stabilized pathway may be favored by the presence of acid‐base pairs or accessible acceptors, regardless of ∙CH_3_ stability.

In addition, Nørskov et al.^[^ ^42^ ^]^ showed that, although two separate descriptors are typically required to account for the two distinct pathways of methane activation, the E_H_ descriptor can still be used as a single predictor of C−H bond activation energies across a wide range of catalyst materials. The rate of methane activation can then be written as:

(1) $$Rate=\vartheta_{\mathrm{motif}}\frac{K_{B}T}{h}\exp\left(\frac{-\Delta G_{a}}{K_{B}T}\right)$$

Here, ϑ_motif_ represents the fraction of available active sites, and the remaining terms express the Arrhenius relation. Given that ∆*G*_a_ can be predicted from the linear relation between *G*_TS_ and *G*_H_, and ϑ_motif_ can be derived from the active oxygen‐site formation free energy (*G*_f_) using the equation below,

(2) $$\vartheta_{\mathrm{motif}}=\frac{\sqrt{P_{O_{2}}}e^{-G_{f}/K_{B}T}}{1+\sqrt{P_{O_{2}}}e^{-G_{f}/K_{B}T}}$$

the methane activation rate can be plotted as functions of *G*_H_ and *G*_f_ (as shown in Figure 2c). Figure 2c shows that all catalyst materials fall on one of two distinct *G*_H_ versus *G*_f_ scaling lines (red and purple lines). Along each line, the dimensionality of the problem is reduced to a single independent descriptor allowing for the creation of a 1D rate volcano (as shown in Figure 2b). Materials located near the top of the volcano offer the best balance between active oxygen‐site coverage and C−H bond activation barrier, making them the most promising catalysts for methane activation. Specifically, the authors have identified oxygen‐promoted IrO_2_ and Cu‐exchanged zeolite surfaces to be near the top of the volcano, which is in agreement with previous experimental studies on IrO_2_(110),^[^ ^47^ ^]^ Cu─O─Cu/MOR,^[^ ^48^ ^]^ bis—oxo‐Cu/MOR,^[^ ^21^ ^]^ and Cu_3_O_3_/MOR^[^ ^19^ ^]^ catalysts.

Note that the volcanoes depicted in Figure 2c,d were obtained based on the assumption that active oxygen sites form under the atmospheric pressure of oxygen. As a result, they are not suitable for identifying highly active electro‐catalysts for methane activation in aqueous electrolytes where ppm levels of dissolved oxygen can be used as an oxidant. Furthermore, electro‐catalytic methane oxidation presents an additional challenge in that, although active oxygen sites/species form readily on the catalyst surface, they can be electrochemically further oxidized to oxygen via OER decreasing the oxygen species coverage and methane oxidation faradaic efficiency. These limitations and how to overcome them by employing carbonate ions as the oxidant will be discussed in more detail in Section 3.

### 2.2 Challenges and Potential Solutions in Electrocatalytic Methane Oxidation

#### 2.2.1 Stability and Durability

Electrocatalytic methane oxidation faces several challenges that hinder its widespread implementation.^[^ ^49^ ^]^ In addition to addressing low activity and selectivity, the stability and durability of catalysts are essential factors to consider for electrocatalytic methane oxidation. The conversion of methane to valuable products using electrocatalysis relies on the efficiency and longevity of the catalyst materials. Unfortunately, the harsh operating conditions, such as high temperatures, corrosive electrolytes, and potential cycling, often lead to catalyst degradation and loss of activity over time. The development of stable and durable catalysts is crucial for the successful implementation of electrocatalytic methane oxidation technologies, enabling long‐term operation, reduced maintenance costs, and improved overall performance. To address these challenges, researchers have been exploring novel catalyst materials, surface modifications, and advanced synthesis techniques to enhance stability and durability, aiming to unlock the full potential of electrocatalytic methane oxidation.

Solid oxide fuel cells (SOFCs) have been extensively investigated for electrocatalytic methane oxidation. Several anode materials have been reported, demonstrating reasonable power density at higher‐temperature SOFCs.^[^ ^38^ ^]^ Early efforts focused on ceramic‐metal composite anodes such as Ni‐YSZ‐CeO_2_, Cu‐YSZ‐CeO_2_, and Cu‐ or Sm‐doped CeO_2_ catalysts, which exhibited activity toward electrocatalytic methane oxidation and remained stable during prolonged operation with dry CH_4_.^[^ ^50^, ^51^ ^]^ Interconnect materials, known for their conductivity and stability, were suggested as suitable candidates for efficient and long‐term use. For example, doping lanthanum chromite with Pd‐Ni/LSCr was investigated for electro‐oxidation of dry methane, achieving steady current generation of 350 mA cm^−2^ and power densities of 150 and 360 mW cm^−2^ at 800 and 900 °C, respectively.^[^ ^52^ ^]^ Further advancements included the use of a different anode configuration, Ni/SDC (Sm_0.2_Ce_0.8_O_2_), where a thin porous layer of SDC was coated on Ni pores.^[^ ^53^ ^]^ This modification led to improved electro‐catalytic activity, reduced carbon deposition compared to Cu‐ceria cermet, and achieved high power densities ranging from 65 to 248 mW cm^−2^ at operational temperatures of 750–800 °C. Compared to conventional Ni/YSZ anodes, this modified configuration resulted in a performance improvement of up to 20–25% with comparable durability of 180 hours. The enhanced performance was attributed to the high ionic conductance, increased active sites, and surface modification with SDC on the Ni anode, which provided protection against degradation and prevented carbon precipitation. These findings suggest that altering the anode configuration can be an effective strategy for enhancing electrocatalytic methane oxidation activity. Overall, high‐temperature SOFCs offer various anode materials for electrocatalytic methane oxidation, demonstrating considerable stability and durability.

#### 2.2.2 Mass Transport Limitations

Next, efficient mass transport of reactants to and products from the catalyst surface is crucial for achieving high‐performance methane oxidation. While aqueous electrolyte cells are useful for rapid catalyst evaluation, they fall short of achieving the high current densities required for industrial processes (200−1000 mA cm^−2^) due to poor mass transfer of methane dissolved in the electrolyte. It is worth noting that non‐polar organic solvents, such as cyclohexane, benzene, and toluene, can enhance methane solubility in liquid electrolytes. However, these electrolytes are more susceptible to oxidation than methane itself. Prajapati et al.^[^ ^54^ ^]^ investigated the faradaic efficiency of methane oxidation reaction on TiO_2_, IrO_2_, and PbO_2_ catalysts at different applied potentials in a neutral pH phosphate buffer electrolyte. They observed that the faradaic efficiency initially increased with increasing applied potential and then exhibited a decrease. The decrease in faradaic efficiency at higher applied potentials suggests the presence of mass transfer limitations affecting the methane oxidation reaction.

To overcome these limitations, the incorporation of a gas diffusion electrode (GDE) with a porous structure in a three‐electrode flow‐cell can greatly enhance the electrolytic oxidation of methane by establishing a triple‐phase boundary of gas–liquid–solid. Rocha et al.^[^ ^55^, ^56^ ^]^ investigated the utilization of a GDE configuration for methane oxidation. They employed a similar cell design previously reported for oxygen reduction to hydrogen peroxide, using a carbon/PTFE GDE consisting of mixed TiO_2_/RuO_2_/V_2_O_5_.^[^ ^57^ ^]^ In their study, the incorporation of an optimal amount of V_2_O_5_ (5.6%) resulted in a doubled total faradaic efficiency of 57% at 2.0 V versus SCE. The authors concluded that the adoption of a GDE, enabling continuous methane feeding to the cell, enhances the selectivity for methanol even at low current densities. Note that the GDE provides several advantages for electrocatalytic methane oxidation, i.e., the porous nature of the GDE enables the efficient diffusion of methane and oxygen through the electrode, ensuring a sufficient supply of reactant gases to the catalyst surface. The inclusion of a GDE addresses the mass transport limitation associated with the low solubility of methane, thereby improving overall performance. Finally, the conductive substrate of the GDE ensures efficient electron transfer between the catalyst and the external circuit, enabling the electrochemical oxidation of methane. Overall, the design and optimization of the GDE structure, along with the choice of catalyst material, are crucial considerations in achieving enhanced methane oxidation performance in electrochemical systems.

Similarly, membrane‐electrode assemblies (MEAs) offer an alternative means to overcome these challenges. MEAs can be either fully vapor‐fed or have a liquid electrolyte solution (e.g., KOH) fed on one or both sides of the cell, providing pH regulation and optimizing the microenvironment around the catalyst.^[^ ^58^, ^59^ ^]^ Furthermore, moderating the cell temperature or using thinner membranes enhances the mass transport of methanol away from the electrode surface which can help suppress the complete oxidation of products.^[^ ^60^, ^61^, ^62^ ^]^ Improved methanol transport can be achieved through electrode surface modifications and optimized reactant gas flow. The electrochemical studies on methane oxidation have been conducted using both aqueous and nonaqueous electrolyte cells, known as half‐cells, as well as membrane‐electrolyte assemblies, referred to as full‐cells, which offer distinct advantages. MEAs are particularly advantageous as they enable achieving high current densities (> 100 mA cm^−2^) necessary for commercially viable applications. However, the performance of these systems is influenced by the mode of operation, the tested microenvironment (acidic or alkaline), and the type of membrane employed (polymer or ceramic). Despite advancements, the main challenge in the field remains the discovery of efficient electrocatalysts. Therefore, enhancing catalyst design and architecture, such as incorporating hierarchical structures, mesoporous materials, and optimized electrode configurations, holds promise for improving reactant accessibility and alleviating mass transport limitations.

#### 2.2.3 High Overpotentials and Low Energy Efficiency

High overpotentials and low energy efficiency represent another critical challenge in electrocatalytic methane oxidation. As mentioned earlier, the oxidation of methane typically necessitates the application of high potentials to drive the electrocatalytic reaction, resulting in increased energy consumption and reduced overall energy efficiency. To address this issue, it is crucial to develop catalysts that can operate at lower overpotentials or explore innovative approaches such as coupling methane oxidation with other electrochemical processes. One promising technique is the combination of photocatalysis and electrocatalysis, which can be achieved through the utilization of a photoelectrode (using either a photoelectric or photoelectrochemical (PEC) approach). This integrated approach has the potential to provide additional energy to fulfill the energy requirements for breaking the inert C−H bonds in methane.

PEC cells present an alternative approach for methane activation by combining the advantages of photocatalysis and electrocatalysis. These systems utilize an n‐type semiconductor on a conductive substrate as a photoanode, and an external circuit enables a current loop between two electrodes in an aqueous electrolyte. Under electrolytic conditions, the photoexcited charge transfer reactions can be accelerated, potentially stabilizing the reaction intermediate species on the catalyst surface and influencing the selectivity of the products. The design of a photoelectrode enables the simultaneous coupling of solar and electrical energy, overcoming the limitations of both photocatalysis and electrocatalysis. This approach addresses the challenges faced by each method individually, as effective oxidation potential cannot be obtained solely through photoexcitation, and the generation of catalytically active sites is limited with electricity alone. Therefore, PEC cells hold significant promise for the activation and conversion of methane.

In recent practical applications, Wang et al.^[^ ^63^ ^]^ developed a selective conversion process of methane to CO using a PEC method. The methane oxidation took place at the interface of the photoanode and electrolyte under room temperature conditions with light irradiation and applied potential. Amano et al.^[^ ^64^ ^]^ also reported the conversion of methane to ethane on a WO_3_ photoanode in a gas‐phase photoelectrochemical system. The process occurred under the irradiation of a 453 nm LED at room temperature, and it was accompanied by the production of H_2_ on the cathode with an ≈100% Faraday efficiency. These recent studies demonstrate the potential of PEC cells for efficient and selective methane activation and highlight their applicability in practical scenarios.

#### 2.2.4 Scalability and Cost‐Effectiveness

Finally, addressing scalability and cost‐effectiveness poses additional challenges in partial electrocatalytic methane oxidation. Achieving scalable production of catalyst materials and integrating them into practical electrochemical systems at a reasonable cost is essential for commercial viability. Exploring scalable synthesis methods, efficient catalyst deposition techniques, and cost‐effective catalyst materials, including abundant and earth‐abundant elements, can address the scalability and cost challenges. Importantly, methane oxidation to desired products through the electrocatalytic pathway on an industrial scale has demonstrated potential economic viability, as indicated by recent techno‐economic analyses (TEA). These analyses consider factors such as device manufacturing costs, carbon and energy efficiencies, and product purification and distribution costs associated with the electrocatalytic process. To evaluate the performance of each process for a desired product, computer‐based simulations are essential. Numerous TEA have focused on methanol production. Ehlinger et al.^[^ ^65^ ^]^ conducted a sensitivity analysis of a methanol plant to assess the influence of shale gas prices and methanol selling prices on overall profitability. They also estimated a price differential of $0.73/MMBTU between utilizing shale gas at the wellhead and natural gas in a methanol plant, mainly attributable to impurities in shale gas. In another TEA, Julian‐Duran et al.^[^ ^66^ ^]^ investigated the economic and environmental impacts of various reforming technologies in a methanol plant. One alternative was combined reforming, which involved utilizing CO_2_ as a feedstock for syngas production. They observed that conventional reforming options (e.g., partial oxidation of methane, auto‐thermal reforming, and steam reforming) exhibited higher profitability compared to combined reforming, although combined reforming was deemed the most environmentally sustainable choice. Very recently, Alsuhaibani et al.^[^ ^67^ ^]^ demonstrated that reducing the reaction pressure in a methanol production reactor using shale gas resulted in substantial profit improvements for a plant with a capacity of 2.1 × 106 tons per year. Therefore, based on a techno‐economic standpoint, methane oxidation to desired products through electrocatalysis on an industrial scale is anticipated to be economically viable.

### 2.3 Role of Different Oxidants in Electrocatalytic Methane Oxidation

**Figure 3:**
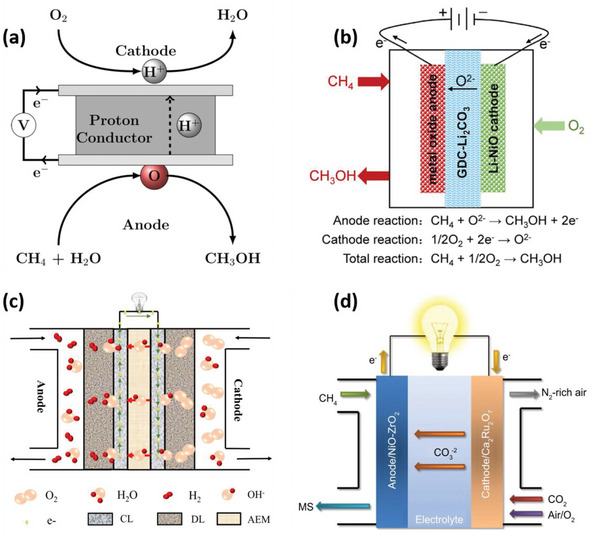
Schematics of an electrochemical cell for the overall conversion of methane to methanol using different oxidants. a) H_2_O,Reproduced with permission.^[^ ^89^ ^]^ Copyright 2016, American Chemical Society. b) O^2−^,Reproduced with Permission.^[^ ^38^ ^]^ Copyright 2018, Elsevier. c) OH^−^,Reproduced with permission.^[^ ^90^ ^]^ Copyright 2018, Elsevier. d) CO_3_^2−^ Reproduced with permission.^[^ ^24^ ^]^ Copyright 2013, IOP Publishing, Ltd.

Methane can be electrochemically oxidized to methanol in either the liquid at low temperature or the gas phase at high temperature.^[^ ^12^, ^68^, ^69^, ^70^ ^]^ However, high‐temperature gas‐phase procedures often result in limited methanol yield due to methanol oxidation to CO_x_. Additionally, catalyst deactivation seems inevitable due to coking, oxidation, and sulfur poisoning from crude CH_4_ input. In contrast, low‐temperature liquid‐phase procedures utilizing oxidants such as NO/N_2_O^[^ ^71^, ^72^, ^73^ ^]^ O_2_^[^ ^74^, ^75^, ^76^, ^77^ ^]^ and H_2_O_2_^[^ ^8^, ^18^, ^78^, ^79^ ^]^ are preferable to replace energy‐intensive processes. The choice of oxidant and its concentration can significantly impact the reaction efficiency and product selectivity. The optimal concentration of the oxidant will depend on the specific conditions of the reaction and the desired products.^[^ ^80^ ^]^ Oxidants also significantly impact the onset potential of methane conversion and the primary oxidation products.^[^ ^81^, ^82^, ^83^, ^84^, ^85^ ^]^ However, a complete discussion on the role of oxidants in thermal partial oxidation is beyond the scope of this review. In electrochemical partial methane oxidation, oxidants play a crucial role in generating active oxygen species for initial methane oxidation. **Figure** **3** shows the schematics of the electrochemical cell concept for the overall conversion of methane to methanol utilizing various oxidants. **Table** **1** highlights several reports in the literature on the oxidants employed in electrochemical partial methane oxidation.

**Table 1:** A list of the most significant reports on electrochemical partial methane oxidation using various oxidants.

Year	Oxidant	Catalyst (Anode;Cathode)	Cell type	Products	Selectivity/Efficiency/Rate	Reference.
2013	H_2_O	TiO_2_/RuO_2_/V_2_O_5_;PTFE	GDE	CH_3_OH, HCHO, HCOOH	57%	[56]
2018	H_2_O	TiO_2_	GDE	CO, CO_2_	81.9%	[63]
2010	H_2_O	TiO_2_/RuO_2_;PTFE	GDE	CH_3_OH	30%	[55]
2011	H_2_O	V_2_O_5_/SnO_2_;Pt/C	Low‐Temperature FC	CH_3_OH	88.4%	[60]
1991	O_2_	Au, glassy carbon, Hg, Cu	GDE	CH_2_O, CH_3_OH CO, and CO_2_	Rate: 1.5 × 10^−3^ mol cm^−2^ h^−1^	[99]
2009	O_2_	Ni(OH)_2_/MWCNTs	Conventional three‐electrode cell	CH_3_OH	—^a)^	[100]
2010	O_2_	CuO_X_ –PdAu/C	Fuel cell Reactor	CH_3_OH, CO_2_	≈100%	[75]
2008	O_2_	PdAu/C	Fuel cell Reactor	CH_3_OH, CO_2_	60%	[74]
2021	OH^−^	Ni(OH)_2_‐NiOOH/Ni Foam Anode	Hall Cell (TEC)	CH_3_OH	—^a)^	[101]
2019	OH^−^	Pd/C Anode, Pt/C Anode, Ni/C Anode	Full Cell (HEM)	CH_3_OH	—^a)^	[92]
2015	OH^−^	MO_X_ Anode (M = Ni, Co, Cu, Ag, Pt, Au, Ce, Pb, Fe, Mn, Zn, or combinations)	Full Cell (HEM)	CH_3_OH, CO_2_	—^a)^	[102]
2014	OH^−^	MO_X_ Anode (M = Mn, Fe, Ni, Os, or Pt)	Full Cell (HEM)	CH_3_OH	—^a)^	[103]
2019	CO_3_^2−^	ZrO_2_/NiCo_2_O_4_	Single‐compartment three‐electrode	CH_3_CH_2_CH_2_OH, CH_3_CH(OH) CH_3_, CH_3_CH_2_COOH, CH_3_COCH_3_, CH_3_COOH	47.5%^b)^	[96]
2017	CO_3_^2−^	Co_3_O_4_‐ZrO_2_	Single‐compartment three‐electrode	CH_3_CH_2_CH_2_OH, (CH_3_)_2_CHOH, CH_3_CHO, CH_3_OH, CH_3_CH_2_OH	60%^c)^	[95]
2013	CO_3_^2−^	NiO‐ZrO_2_	MEA	CH_3_OH, HCHO, CO, HCOOH, CH_3_CH_2_OH, CH_3_COOH, CH_3_COCH_3_, CH_3_CHOHCH_3_	—^a)^	[24]
2021	CO_3_^2−^	CuO/CeO_2_	Single‐compartment three‐electrode	CH_3_OH, C_2_H_5_OH, CH_3_COCH_3_	79%	[104]
2023	CO_3_^2−^	NiO/ZnO	Single‐compartment three‐electrode	C_2_H_5_OH, CH_3_OH, CH_3_COCH_3_	81%	[105]

^a)^ Methane was overoxidized to CO and CO_2_, or TOF of methanol is not reported;

^b)^ methane oxidation conversion efficiency;

^c)^ Total production efficiency of 1‐propanol and 2‐propanol;

GDE, Gas Diffusion Electrode; FC, Fuel Cell; MEA, Membrane Electrode Assembly.

Water is widely recognized as a favorable oxidant in the direct thermal oxidation of methane to methanol, owing to its superior ability to promote methanol desorption and stabilize reaction intermediates compared to other oxidants.^[^ ^16^, ^21^, ^86^ ^]^ In electrocatalysis, water is utilized to generate active oxygen species on the catalyst surface, facilitating hydrogen abstraction from methane. However, complete oxidation of water via the OER considerably reduces selectivity and faradaic efficiency of methane conversion. Prior studies have demonstrated that SOFCs can address this issue. In SOFCs, methane and steam (CH_4_(g) + H_2_O(g) → CH_3_OH(g) 2H^+^ +2e^−^) are fed to the anode, and the protons produced at the anode are transported to the cathode by a proton‐conducting membrane. At the cathode, oxygen from air is reduced ($\frac{1}{2}$ O_2_(g) + 2H^+^ +2e^−^ → H_2_O(g)) (Figure 3a). The primary advantage of this approach is the utilization of easily accessible water vapor as an active oxygen source. To date, the most effective system employs V_2_O_5_/SnO_2_^[^ ^60^ ^]^ as the anode catalyst/support and carbon‐supported platinum for the cathode, with Sn_0.9_In_0.1_P_2_O_7_^[^ ^75^ ^]^ serving as the proton conductor, operating at temperatures ranging from 100 to 200 °C. A selectivity of 61% was achieved at a current density of ∼2 mA cm^−2^, with an overall single‐pass product yield of ≈0.3%.

In partial electrochemical methane oxidation, using O_2_ as the oxidizing agent results in a spin‐forbidden reaction, where triplet O_2_ and singlet CH_4_ produce singlet methanol, suggesting that O_2_ is not an efficient proton‐extracting agent under mild conditions.^[^ ^87^ ^]^ In contrast, SOFCs generate active oxygen species (O^2−^ ions) at the cathode, which are transferred to the anode through an ion‐conductive solid oxide electrolyte to activate the C−H bond (Figure 3b). The applied potentials in the cathode split oxygen‐containing molecules, such as gaseous O_2_ or H_2_O to form O^2−^ ions, which are simultaneously transferred to the anode.^[^ ^50^, ^88^ ^]^ Torabi et al. demonstrated that NiO and Li‐doped SrTiO_3_ perovskite could achieve over 90% methanol selectivity as the cathode and anode, respectively, of an SOFC in the temperature range of 300 °C to 600 °C. The presence of O^2−^ in the reaction enhances the selective production of oxygenates. Therefore, using H_2_O or O_2_ as oxidants in SOFC systems offers the unique advantage of directly electrolyzing oxidants to produce oxygen species in the anode, while selectively oxidizing methane to alcohol in the cathode.

In addition to water and oxygen, hydroxyl radicals (OH∙) have also been explored as an oxygen source for methane oxidation (Figure 3c). Nandenha et al.^[^ ^91^ ^]^ used a solid membrane reactor‐PEM fuel cell to partially oxidize methane to methanol at room temperature by feeding CH_4_ and an H_2_O_2_ solution into the cathode. The catalytic layer absorbed methane and H_2_O_2_, producing OH∙ that reacted with methane to produce methanol. Methanol production was highest in the potential range of 600‐400 mV. The effects of OH∙ in electrocatalytic methane oxidation depend on the presence or absence of hydrogen peroxide (H_2_O_2_). In the presence of H_2_O_2_, OH∙ generated from the reaction between H_2_O_2_ and the catalyst play a crucial role in enhancing methane oxidation. The combined presence of OH∙ and H_2_O_2_ accelerates the kinetics of methane oxidation by facilitating the breaking of C−H bonds. Conversely, in the absence of H_2_O_2_, the availability of OH∙ for methane oxidation is reduced, relying on alternative reaction pathways involving water molecules or other reactive species. This absence of H_2_O_2_ and limited OH∙ availability leads to slower reaction rates, hindering the oxidative capability and efficiency of methane oxidation. Overall, H_2_O_2_ enhances the generation and reactivity of OH∙, resulting in more efficient electrocatalytic methane oxidation. Alkaline anion exchange membrane fuel cells (AAEMFCs) have also been utilized to generate OH^−^ ions, which are transported using an ion‐conductive membrane from the cathode to the anode.^[^ ^90^ ^]^ Santos et al.^[^ ^92^ ^]^ investigated Pt/C, Pd/C, and Ni/C as anode materials for methane oxidation using an AAEMFC operating at room temperature. FTIR data indicated that methane was converted into organic molecules such as methanol and formate at different potentials. The highest conversion efficiency was reported to be >20% at 0.3 V using a Pt/C catalyst. Pd/C showed a maximum conversion of 17.5% at 0.15 V, associated with the formation of a thin layer of PdO on the catalyst surface. Surprisingly, Ni/C could activate methane with no applied potential.

Carbonate (CO_3_^2−^) ions are an attractive oxidizing agent due to their ability to provide a charged oxygen atom through the CO_3_^2−^ ↔ CO_2_ + O_2_ reaction under mild conditions. Spinner et al. were the first to demonstrate that a ZrO_2_ and NiO composite could activate and partially oxidize methane to methanol through oxygen insertion by using CO_3_^2−^ as an oxidizing agent (Figure 3d).^[^ ^24^ ^]^ Carbonate anions were generated at the cathode by the reaction of CO_2_ and O_2_ using either calcium‐ruthenium oxide or Pt/C electrodes.^[^ ^93^, ^94^ ^]^ Since then, composites of ZrO_2_ and metal oxide nanoparticles have gained significant attention for direct methane oxidation. For example, ZrO_2_ composites with Co_3_O_4_,^[^ ^95^ ^]^ NiCo_2_O_4_,^[^ ^96^ ^]^ and CuO_2_^[^ ^97^ ^]^ have shown better catalytic activity toward partial methane oxidation than the composite containing NiO. It has been shown that methanol is only an intermediate and is further oxidized to produce higher alcohols and carboxylic acids due to CO_3_^2−^ as an oxidizing agent. Prior studies have revealed the existence of competition between methane oxidation and the OER.^[^ ^97^, ^98^ ^]^ It was unclear whether these reactions are involved in the same mechanistic pathway or are parallel reactions. This review article will also present our perspectives on circumventing competition with OER using CO_3_^2−^ ions. In summary, the choice of the oxidant significantly impacts the efficiency and selectivity of the reaction, and oxidants play a critical role in electrochemical partial methane oxidation by providing the active oxygen species for initial methane oxidation, which can then be further processed to produce various alcohols and chemicals.

### 2.4 Effect of Reaction Conditions on Electrocatalytic Partial Methane Oxidation: Electrolyte versus Pressure versus Temperature versus Applied Potential

The electrochemical partial oxidation of methane to methanol is influenced by several factors, such as the electrolyte, pressure, temperature, and applied potential. Optimizing these parameters can improve conversion efficiency, selectivity, and product yield. However, developing efficient and stable catalysts for partial methane oxidation remains a significant challenge. This section provides an overview of the research on the effects of electrolyte, pressure, temperature, and applied potential on the process, enhancing our understanding of how these parameters contribute to the development of stable and efficient catalysts.

#### 2.4.1 Electrolyte

The choice of electrolyte can significantly impact the electrochemical partial oxidation of methane to methanol. While studies have primarily investigated methane electrochemical oxidation in aqueous electrolytes, research on solid‐state electrolytes, ionic liquids, and organic electrolytes has also been conducted over the years. In liquid‐phase electrolyzers, the presence of liquid electrolytes is crucial for promoting ion transport and creating a favorable reaction environment. Liquid electrolytes offer the advantage of controlling the kinetics of methane electrocatalysis at the electrode/electrolyte interface by adjusting the pH. However, the extremely low solubility of methane in aqueous solutions (<22.7 mg∙L^−1^) makes it challenging to achieve high product yields in conventional electrocatalytic systems operating in acidic, alkaline, or neutral media.^[^ ^106^, ^107^, ^108^ ^]^ While non‐polar media can improve methane solubility in liquid electrolytes, these organic solvents are more readily electro‐oxidized before methane. To overcome this challenge, a porous gas diffusion electrode has been used to significantly increase the number of gas‐liquid‐solid interfaces, thereby favoring efficient electrocatalytic methane oxidation.^[^ ^56^ ^]^ **Table** **2** summarizes the representative electrolytes with electrocatalysts, reaction products, and current densities. It can be observed that the electrochemical oxidation of methane in an aqueous medium at noble metal electrodes ultimately leads to complete oxidation to CO_2_. However, contrary to expectations, Santos et al.^[^ ^92^ ^]^ demonstrated that methane can be effectively converted to methanol on a Pt/C electrocatalyst by 20% at 0.3 V cell voltage using an AAEMFC. This improvement was attributed to a reduction in proton availability near the reaction interface in the alkaline medium, facilitating methanol production.

**Table 2:** Various aqueous electrolytes used in electrochemical systems for methane oxidation.

Electrolyte	Catalyst	Products	Current density [mA cm^−2^]	Reference
H_2_SO_4_	Pt, Pd on C	CO_2_	0.8	[110]
NaCl+ H_2_SO_4_	Pt Salts	CH_3_OH, CH_3_Cl, HCOOH, CO_2_	1.1	[111]
H_3_PO_4_	Pt‐black	CO_2_	50^a)^	[112]
H_3_PO_4_	Pt‐Raney/Au	CO_2_	85^a)^	[113]
HClO_4_	Pt, Ru, Pd Au	CO_2_	—^b)^	[114]
KCl	Pt	CH_3_OH, CH_3_Cl, CH_2_Cl_2_, CHCl_3_, CCl_4_ (total FE:18%)	6.16	[115]
Na_2_CO_3_	NiO‐ZrO_2_	CH_3_OH, HCHO, CO	21	[24]
Na_2_CO_3_	Co_3_O_4_‐ZrO_2_	CH_3_OH, HCHO, C_2_H_4_O, C_3_H_8_O, C_3_H_6_O (total FE:47%)	4.7	[96]
Na_2_SO_4_	TiO_2_‐RuO_2_/PTFE	CH_3_OH, HCHO, HCOOH	13	[55]
KOH	MOx (M = transition metals)	CH_3_OH, CO_2_	10	[102]

^a)^ (mA cm^−2^ at E = 0.5 V (SHE));

^b)^ Methane was overoxidized;

to CO and CO_2_, or current density is not reported.

Qiao et al. investigated Ni(OH)_2_‐modified Ni electrodes in 1 mol L^−1^ NaOH solution for electrochemical methane oxidation.^[^ ^100^ ^]^ The cyclic voltammetry of the Ni electrode resulted in the creation of Ni(OH)_2_ on the surface. Subsequently, at a potential sweep rate of 20 mV s^−1^ in a solution of 1.0 mol L^−1^ NaOH, methane was absorbed on a Ni‐oxy‐included surface and converted into methanol. This established that the electrolyte oxidizes the catalyst surface, causing electronic rearrangement, which generates lattice oxygen species for methane oxidation. Similarly, Spinner et al. developed a NiO/ZrO_2_ bifunctional catalyst by using CO_3_^2−^ ions as an oxygen‐donating species.^[^ ^24^ ^]^ Interestingly, CO_3_^2−^ based electrolytes effectively oxidize methane through its charged oxygen atom, resulting in a lowered reaction barrier and accelerated kinetics for the methane oxidation reaction.^[^ ^24^, ^108^, ^109^ ^]^ Therefore, CO_3_^2−^ based electrolytes outperform other electrolytes in alkaline‐based systems.^[^ ^95^, ^96^, ^97^ ^]^

The stability of catalyst ions during the catalytic cycle of methane oxidation using Pt and Pd salts has been reported in acidic media.^[^ ^111^, ^116^ ^]^ To avoid the stoichiometric usage of expensive Pt^IV^ oxidant with aqueous Pt salts (Pt^II/IV^) in a concentrated acid medium (0.5 _m_ H_2_SO_4_), Kim et al.^[^ ^111^ ^]^ used the Shilov catalytic system. This strategy maintained the Pt^II/IV^ ratio throughout the Pt^II^‐catalyzed methane oxidation cycle, resulting in a stable methane oxidation catalysis with 70% selectivity for methanol. It should be noted that methane molecules compete with electrolyte anions for adsorption sites, and the nature of the electrolyte anions influences methane adsorption. As a result, CH_4_ adsorption is highest in HClO_4_, followed by H_3_PO_4_ and H_2_SO_4_ solutions. The choice of electrolyte can affect the performance of the electrochemical cell by influencing the electrode materials' oxygen ion conductivity, surface reactivity, and stability. Furthermore, using a suitable electrolyte for a particular system can also impact the selectivity and yield of methanol.

#### 2.4.2 Pressure

The electrochemical partial oxidation of methane to methanol can also be influenced by the pressure of the reactants. Increasing the pressure has two significant advantages: 1) it increases the methane solubility in an aqueous solution, and 2) it increases the reaction rate due to the increased collision frequency between the reactants and the electrode surface, resulting in higher oxidation current densities. However, high pressures can also lead to increased mass transport limitations and reduced gas‐phase mass transfer. Additionally, adjusting the partial and absolute pressure of methane changes the product selectivity by altering the relative surface coverage, adsorbed oxidant binding energies, and methane oxidation intermediates. Therefore, determining an optimal pressure range is crucial for improved catalytic activity. Nonetheless, several investigations have established pressured systems for electrochemical partial methane oxidation.^[^ ^111^, ^116^ ^]^

**Figure 4:**
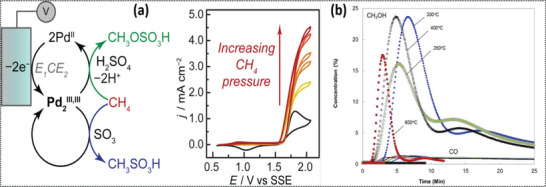
a) The effect of increasing CH_4_ pressure (0 to 500 psi) on functionalization by an electrogenerated Pd_2_^III,III^ species. CVs (20 mV s^−1^ scan rate) of PdSO_4_ (≈23 mM) in concentrated H_2_SO_4_. Reproduced with permission.^[^ ^117^ ^]^ Copyright 2020, American Chemical Society. b) NiO/La‐doped SrTiO_3_ at various temperatures as a function of time. Reproduced with permission,^[^ ^118^ ^]^ Copyright 2016, IOP Publishing.

O'Reilly et al.^[^ ^116^ ^]^ investigated the effect of pressure on the electrochemical partial oxidation of methane to methanol. Their study demonstrated the rapid electrochemical functionalization of methane to methanol precursors (i.e., methyl bisulfate (CH_3_OSO_3_H) and methane sulfonic acid (CH_3_SO_3_H) using the electro‐oxidation of Pd^II^SO_4_ in concentrated sulfuric acid at high methane pressure (**Figure** **4**a). This reaction system is identical to the thermocatalytic Periana system, which explains the same chemistry in a similar reaction media using molecular catalysts (2,2′‐bipyrimidyl)platinum(II) dichloride. Increasing the methane pressure to 100 psi in the electrochemical cell led to the anodic peak at 1.82 V, resulting in higher current densities of up to 500 psi. Similar behavior of direct proportionality of pressure with current density was observed when the reaction temperature was lowered to 100 °C. However, the anodic current decreased beyond 1.5 V due to thweakening of the Pd_2_^III, III^ back‐reduction peak. These findings suggest that determining the optimal pressure for a given electrochemical partial methane oxidation reaction requires careful consideration of the reaction kinetics and product selectivity.

#### 2.4.3 Temperature

One of the distinct benefits of employing electrocatalytic partial methane oxidation is the ability to maintain ambient temperature throughout the process. Nevertheless, temperature remains a crucial parameter with a substantial impact on the rate and selectivity of the electrochemical partial methane oxidation process for methanol production. The kinetics of direct electrochemical oxidation under mild conditions are typically sluggish due to the high C−H bond activation energy. Increasing the reaction temperature typically leads to an increase in the reaction rate due to the increased mobility of the reacting species. However, high temperatures can also result in increased electrode degradation and material instability. To date, partial electrochemical oxidation of methane has been widely investigated at room temperature in liquid‐phase electrolytes. Hibino et al. investigated the effect of both temperature and applied potential on the partial electrochemical oxidation of methane to methanol using a confined fuel cell‐type reactor (proton‐conducting SOFCs (P‐SOFC) systems).^[^ ^60^ ^]^ Initially, a Pt/C anode was fed with a mixture of methane and water vapor to carry out the reaction. The methane concentration decreased as the reaction temperature increased from 50 to 250 °C, resulting in an increase in the current density. As the temperature increased, the two byproducts, O_2,_ and CO_2_ decreased and increased, respectively. Furthermore, non‐noble metal oxide catalysts with non‐carbon supports were investigated to increase product yield toward CH_3_OH, achieving a 50% increase in methanol selectivity and a 61.4% increase in Faradaic efficiency on V_2_O_5_/SnO_2_ at 100 °C. Finally, the authors demonstrated that the active oxygen species produced by the anodic polarization of water vapor over V^4+^ sites at potentials of +900 mV allow methane oxidation to methanol.

The fundamental shortcoming of the earlier systems is that they require >1 V of applied potential.^[^ ^89^ ^]^ In a subsequent study, a similar setup was used with an oxide‐conducting solid oxide fuel cell (O‐SOFC) operating at temperatures ranging from 300 to 600 °C, which achieved ≈90% methanol selectivity over a NiO/La‐doped SrTiO_3_ perovskite anode (Figure 4b).^[^ ^118^ ^]^ Perovskite/O‐SOFC catalysts facilitate the transfer of intermediate oxygen ions to lattice oxygen ions, implying that lattice oxygen species contribute to higher methanol selectivity. Tomita et al. reported that Pd_8_Au_1_/C catalyst oxidizes methane to methanol and carbon dioxide.^[^ ^74^ ^]^ Formation rates of both gases increase with temperature, but the temperature dependency of CO_2_ is slightly greater than that of CH_3_OH. As a result, decreasing the temperature increases the selectivity toward methanol by 6.03% at 250 °C, 10.8% at 150 °C, and 60.0% at 50 °C. These studies demonstrate that temperature plays a significant role in the partial methane oxidation reaction to methanol, affecting the reaction rate and product selectivity. Therefore, finding the optimal temperature requires careful consideration of the desired product selectivity, reaction kinetics, and other factors.

#### 2.4.4 Applied Potential

The applied potential plays a crucial role in electrocatalytic systems, as it directly influences the thermodynamic landscape of the reaction plane while keeping temperature, pressure, and composition constant. At the electrode/electrolyte interface, changes in the electrochemical potential, driven by the externally applied potential, have a profound impact on the activation barriers for species adsorption, oxidation, and reduction, as well as desorption within the double layer.^[^ ^119^ ^]^ For instance, the V_2_O_5_/SnO_2_ catalyst demonstrated a methanol current efficiency of 61.4% and selectivity of 88.4% at 100 °C. Notably, the methanol concentration demonstrated a pronounced peak at potentials ≈+900 mV across all tested V_2_O_5_ contents, highlighting the significant influence of applied potential on methanol generation. Similarly, Zheng et al.^[^ ^120^ ^]^ conducted an investigation on the electrochemical methane oxidation using ZnO and Rh/ZnO catalysts within a potential range of 2.0 to 2.4 V. Within this applied potential range, the Faradaic efficiencies and production rates of ethanol exhibited a trend of initially increasing and then decreasing for all the Rh/ZnO catalysts. Notably, at 2.2 V, the 0.6% Rh/ZnO catalyst demonstrated the highest ethanol production Faradaic efficiency of 22.5% and the highest ethanol yield rate of 789 µmol·g_cat_^−1^·h^−1^. The reduction in ethanol production rate at more positive potentials was attributed to competition with OER, as well as the overoxidation of ethanol, consistent with previous findings in the literature.^[^ ^96^ ^]^ This observation underscores the importance of carefully considering the electrode potential in optimizing the performance of the catalyst system. Moreover, the applied potential affects the kinetics of electron transport and charge transfer on oxidized surfaces, thereby influencing the rate of oxidative species formation. This effect is observed in processes like water splitting for *O surface generation and the production of initiators for radical activation, both involving electron transfers as electrochemical processes. Kinetic models have been developed to investigate the maximum achievable reaction rate and the minimum potential required for various types of materials, including metals, metal oxides, and MXenes.^[^ ^29^, ^121^ ^]^ By establishing correlations with the descriptors, such as the adsorption energy of *O‐involved intermediates, the methane oxidation activity of metals in electrocatalytic methane oxidation and the methanol generation rate at the theoretical applied potential for oxides and MXenes can be predicted. Among the metals studied, platinum exhibited a high rate of methane oxidation due to its weak *OH and strong *CO adsorption energies. The minimum potential required for Pt was ≈0.96 eV, ensuring resistance against *CO poisoning. In the case of metal oxides and MXenes, a weak *O adsorption energy could enhance the rate of methanol production within the potential range where the OER is not predominant. Hence, applied potential control emerges as a crucial factor for modulating the electrocatalytic methane oxidation rate and selectivity.

## 3 The Promise of Carbonate Ion as the Oxidant for Electrocatalytic Methane‐to‐Methanol Conversion

While we have just discussed the effects of employing different oxidants on the performance of electrocatalytic methane conversion systems, the trend is that many researchers still focus on employing water as the oxidant, similar to traditional low‐temperature electrochemical systems. In this section, we present a brief overview of the mechanistic requirements for high methanol selectivity in electro‐catalytic methane oxidation, limitations of employing water as the oxidant, and discuss differences that arise when CO_3_^2−^ are used as an alternative oxidant.

### 3.1 Mechanistic Requirements for High Methanol Selectivity

Electrochemical methane oxidation to methanol using water as the oxidant generally consists of two stages. The first stage involves the production of active surface oxygen species, such as O* and OH*, from water through electrochemical water oxidation as follows:

(3) $$*+2H_{2}O\to \mathrm{OH}*+H_{2}O+\left(H^{+}+e^{-}\right)$$

(4) $$\mathrm{OH}*+H_{2}O\to O*+H_{2}O+\left(H^{+}+e^{-}\right)$$

Although oxygen species are formed on the catalyst surface for O‐/OH‐assisted methane activation, they can be further electrochemically oxidized to oxygen gas via OER as follows:

(5) $$O*+H_{2}O\to \mathrm{OOH}*+\left(H^{+}+e^{-}\right)$$

(6) $$\mathrm{OOH}*\to *+O_{2}+\left(H^{+}+e^{-}\right)$$

The second stage involves a direct reaction between the adsorbed oxygen species and methane. The theoretically preferred reaction pathway for high methanol selectivity would be as follows:

(7) $$O*+{\mathrm{CH}}_{4}\left(g\right)\to O*•••{\mathrm{CH}}_{4}$$

(8) $$O*•••{\mathrm{CH}}_{4}\to \left(\mathrm{OH}•••{\mathrm{CH}}_{3}\right)*$$

(9) $$\left(\mathrm{OH}•••{\mathrm{CH}}_{3}\right)*\to {\mathrm{CH}}_{3}\mathrm{OH}*$$

(10) $${\mathrm{CH}}_{3}\mathrm{OH}*\to *+{\mathrm{CH}}_{3}\mathrm{OH}$$

Note that none of the reaction steps shown in Equations (7) to (10) are electrochemical reactions. This is crucial for high methanol selectivity as we can avoid the use of high oxidation potential that will promote OER. Interestingly, all of these thermochemical steps are found to be highly favorable with activation barriers <0.7 eV on many oxygen‐promoted catalyst materials such as Fe_2_O_3_ and IrO_2_ (not yet published).

Another important challenge to achieving high methanol selectivity is to prevent the formation of CH_3_O* and CH_3_*. Formation of CH_3_O* via Equations (11) or (12) must be avoided as CH_3_O* cannot be reduced to CH_3_OH* under anodic conditions. CH_3_*, formed via Equations (13), (14), or (15), is prone to further oxidation to CH_2_*, CH*, and C*, leading to the production of other undesirable products like formaldehyde, carbon monoxide, and carbon dioxide. Activation of CH_4_(g) is typically more endothermic than activations of CH_3_*, CH_2_*, and CH*. Under conditions where CH_4_(g) can be activated, CH_3_*, CH_2_*, and CH* can also be deprotonated, leading to the formation of undesired products.

(11) $$O*+{\mathrm{CH}}_{4}\left(g\right)\to {\mathrm{CH}}_{3}O*+\left(H^{+}+e^{-}\right)$$

(12) $$O*{\mathrm{CH}}_{4}\left(g\right)\to {\mathrm{CH}}_{3}O*+H*$$

(13) $${\mathrm{CH}}_{4}\left(g\right)\to {\mathrm{CH}}_{3}*+\left(H^{+}+e^{-}\right)$$

(14) $$*+{\mathrm{CH}}_{4}\left(g\right)\to {\mathrm{CH}}_{3}*+H*$$

(15) $$\mathrm{OH}*+{\mathrm{CH}}_{4}\left(g\right)\to H_{2}O+{\mathrm{CH}}_{3}*$$

As Equation (7) is desirable while Equation (15) is not, atomic oxygen (O*) is the most favored surface oxygen species for methanol production.

Finally, CH_3_OH* desorption (Equation (10)) needs to be more favorable than the deprotonation of CH_3_OH* to CH_2_OH*. To achieve this, water electrolytes must be used to stabilize methanol via solvation.

### 3.2 Limitation of Employing Water as the Oxidant

To achieve highly efficient methane activation via the O‐assisted mechanism, it is essential for O* to be weakly bound (i.e., fairly unstable) on the catalyst surface. This means that the methane oxidation catalyst needs to be relatively oxyphobic. As a result, the OH* or O* formation step (Equations (3) or (4)) becomes the potential determining step compared to the OOH* or O_2_ formation step (Equations (5) or (6)). Therefore, a high oxidation potential must be applied to form O* from water; however, at this high potential, O* is susceptible to complete oxidation via OER.

On the other hand, a high coverage of O* on the catalyst surface is also desired as it would promote CH_3_OH* formation via Equations (16) and (17), while a low coverage of O* would promote the undesired CH_3_* formation via Equation (18). Note that the reaction shown in Equation (17) would be significantly easier than that shown in Equation (19) on most catalyst materials as the latter reaction requires a severe tilting of both CH_3_* and OH* to combine with each other. To achieve a high coverage of O*, the O* formation step (Equation (4)) needs to be, as opposed to the above, more exothermic than the OOH* formation step (Equation (5)).

(16) $$2O*+{\mathrm{CH}}_{4}\left(g\right)\to {\mathrm{CH}}_{3}O*+\mathrm{OH}*$$

(17) $${\mathrm{CH}}_{3}O*+\mathrm{OH}*\to {\mathrm{CH}}_{3}\mathrm{OH}*+O*$$

(18) $$O*+*+{\mathrm{CH}}_{4}\left(g\right)\to {\mathrm{CH}}_{3}*+\mathrm{OH}*$$

(19) $${\mathrm{CH}}_{3}*+\mathrm{OH}*\to {\mathrm{CH}}_{3}\mathrm{OH}*$$

Therefore, in principle, it is quite challenging to attain high coverage of fairly unstable O* on methane oxidation catalysts via electrochemical water oxidation without promoting OER. This limitation represents a fundamental obstacle in using water as the oxidant for electro‐catalytic methane‐to‐methanol conversion.

**Figure 5:**
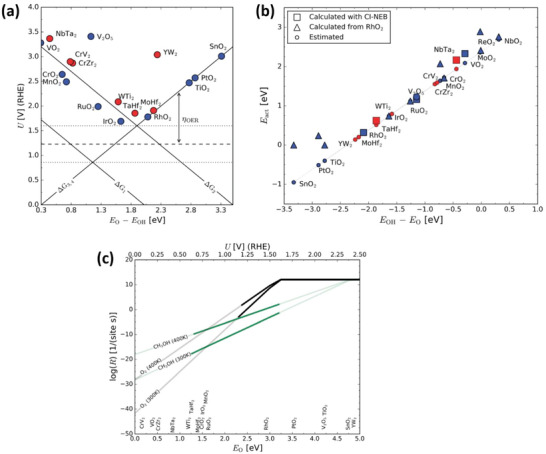
a) Activity volcano plot for the water oxidation reaction, with results shown for rutile transition metal oxides (blue circles) and MXenes (red circles). b) Activation barrier for methane activation versus the descriptor *E*_OH_ − *E*_O_, with blue points representing metal oxides and red points representing MXenes. c) The reaction rates for the OER (black line) and methane oxidation (green line) were plotted at different temperatures as a function of E_O_ and potential. Reproduced with permission.^[^ ^29^ ^]^ Copyright 2018, Royal Society of Chemistry.

Arnarson et al.^[^ ^29^ ^]^ have also explored a similar concept. The authors reported that, although catalyst materials on the weakly binding leg of the OER volcano (refer to **Figure** **5**a) are highly active for methane activation (see Figure 5b). However, considering the availability of O* on the catalyst surface at an increased oxidation potential, catalyst materials closer to the top of the OER volcano, i.e., with intermediate O* binding strength, show the most promising results for methane activation. Consequently, the study concluded that no catalyst materials are capable of producing methanol more efficiently than oxygen, even at an elevated temperature of 400 K (see Figure 5c).

### 3.3 Overcoming the Limitation by Employing Carbonate Ions as the Oxidant

Since a high O* coverage is desired for high methanol selectivity, a rational strategy is to maintain an aqueous environment to stabilize methanol but use a separate oxidant that is strong enough to form a high O* coverage at a relatively low oxidation potential, in order to avoid OER. Interestingly, this approach goes against the conventional thought that weak oxidants such as H_2_O_2_ and N_2_O are beneficial for thermo‐catalytic partial oxidation of methane to methanol. One way to achieve this is by using CO_3_^2−^ as the oxidant. CO_3_^2−^ ions can generate active atomic oxygen on the catalyst surface via the following reactions:

(20) $${\mathrm{CO}}_{3}^{2-}+*\to {\mathrm{CO}}_{3}*+2e^{-}$$

(21) $${\mathrm{CO}}_{3}*\to O*+{\mathrm{CO}}_{2}\left(g\right)$$

Equations (20) and (21) represent the electrochemical adsorption of a carbonate ion to CO_3_*, and the thermochemical dissociation of the adsorbed CO_3_* into O* and CO_2_(g), respectively.

Note that the former reaction occurs via a two‐electron transfer. This indicates that the reaction can become exothermic at low oxidation potential as the reaction free energy (in electronvolts) decreases twice the applied potential. As a result, the complete oxidation of the surface oxygen species to oxygen gas via OER can be avoided, which was a persistent issue when water was used as the oxidant. On the other hand, the latter reaction is generally spontaneous on most catalyst materials since CO_3_* that binds to the surface via the bidentate structure is not very stable unless the two binding sites are very close to each other. Our calculations confirm that the dissociation barriers on Fe_2_O_3_ and IrO_2_ are indeed <0.6 eV (not yet published). Thus, using carbonate ions as the oxidant makes it possible to form a highly O*‐covered surface at low oxidation potential, enabling the methanol oxidation to proceed through the theoretically preferred reaction pathway for high methanol selectivity (Equations (7) to (10)).

### 3.4 Experimental Validation

**Figure 6:**
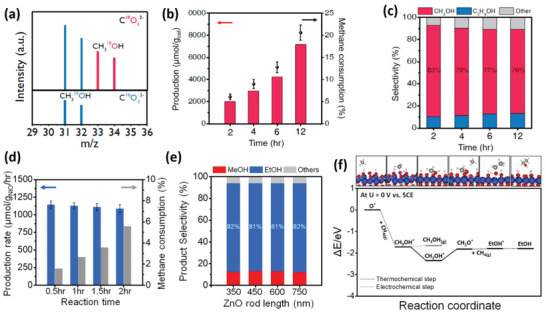
a) Gas chromatography‐mass spectrometry (GC‐MS) results obtained for the electro‐catalytic methane oxidation on CuO/CeO_2_ in an aqueous electrolyte containing ^18^CO_3_^2−^ (top panel). The proportion of CH_3_OH containing ^18^O and ^16^O is calculated as 38% and 62%, respectively. Typical MS for CH_3_OH (bottom panel). b) Production of CH_3_OH and consumption of CH_4_ according to reaction time. c) Selectivity for CH_3_OH and other oxygenates for each reaction time. Reproduced with permission.^[^ ^104^ ^]^ Copyright 2021, American Chemical Society. d) Ethanol production rates during 0.5, 1, 1.5, and 2 h reactions with 600 nm NiO/ZnO nanorod catalysts. e) selectivity for oxygenates with NiO/ZnO nanorod catalysts of various lengths. f) Free energy diagram of the reaction coordinate for methane‐ethanol conversion.^[^ ^105^ ^]^ The cartoons are atomic configurations corresponding to each reaction coordinate; the Ni atom on the surface corresponds to the site where the adsorption of carbonate oxygen forms O*. Reproduced with permission.^[^ ^105^ ^]^ Copyright 2023, Elsevier.

Recently, our experimental collaborators, Moon et al. have shown that CO_3_^2−^ ions can indeed provide active surface oxygen species for efficient electro‐catalytic methane conversion to alcohol on CuO/CeO_2_,^[^ ^67^ ^]^ NiO/ZnO,^[^ ^68^ ^]^ Fe_2_O_3_ and IrO_2_ (not yet published). In particular, **Figure** **6**a shows the results of gas chromatography‐mass spectrometry (GC‐MS) analysis for the products produced during electro‐catalytic methane oxidation on CuO/CeO_2_ in an aqueous electrolyte containing C^18^O_3_^2−^ ions.^[^ ^67^ ^]^ The methanol produced contained ^18^O from the carbonate ion, indicating that methane was activated by the oxygen species from the carbonate ion. This resulted in a maximum production rate of 752.9 µmol g_cat_^−1^ h^−1^ with a methanol selectivity of 79% (see Figure 6b,c), and the earliest onset potential of 0.6 V versus SCE.^[^ ^67^ ^]^

Moreover, Moon et al. have discovered that ethanol can be directly produced from methane by adopting the same concept of using CO_3_^2−^ as the oxidant for the NiO/ZnO nanorod catalyst.^[^ ^68^ ^]^ The average production rate throughout the two‐hour reaction was 1084 mol g_NiO_^−1^ hr^−1^, and the conversion rate remained stable with a reduction of under 5% (Figure 6d). The ethanol selectivity was reported to be 81–82% regardless of the nanorod lengths (Figure 6e). Compared to recently reported electro‐catalysts such as CuO/CeO_2_,^[^ ^104^ ^]^ NiO/ZrO_2_,^[^ ^24^ ^]^ Rh‐ZnO,^[^ ^120^ ^]^ ZrO_2_‐NT/Co_3_O_4_,^[^ ^122^ ^]^ and TiO_2_/RuO_2_/V_2_O_5_,^[^ ^56^ ^]^ the NiO/ZnO nanorod showed significantly enhanced production rates with exceptionally high selectivities, which can be attributed to the CO_3_^2−^. The minimum energy pathway for ethanol production was also identified based on density functional theory (DFT) calculations. The pathway involved methane interacting with O* from a carbonate ion to form CH_3_OH*, deprotonation of CH_3_OH* to CH_2_O*, and methane coupling with CH_2_O* to form ethanol (see Figure 6f). These experiments validate our theoretical perspective on overcoming the fundamental limitation in electrochemical methane oxidation to alcohol using carbonate ions as the oxidant.

### 3.5 Addressing the Environmental Concern

One concern with using CO_3_^2−^ as the oxidant for electro‐catalytic methane conversion to alcohol is the potential for significant CO_2_ emissions from CO_3_^2−^ via Equation (21). One solution to this problem is to convert the produced CO_2_ back to CO_3_^2−^ by controlling the electrolyte pH. The idea draws from the mechanism of controlling the pH balance of blood in our body using bicarbonate (HCO_3_^−^) as a buffer, as shown in the following equations:

(22) $${\mathrm{CO}}_{2\left(\mathrm{aq}\right)}+H_{2}O⇌H_{2}{\mathrm{CO}}_{3}$$

(23) $$H_{2}{\mathrm{CO}}_{3}⇌{\mathrm{HCO}}_{3}^{-}+H^{+}$$

(24) $${\mathrm{HCO}}_{3}^{-}⇌{\mathrm{CO}}_{3}^{2-}+H^{+}$$

(25) $${\mathrm{CO}}_{2}+{\mathrm{OH}}^{-}⇌{\mathrm{HCO}}_{3}^{-}$$

(26) $${\mathrm{HCO}}_{3}^{-}+{\mathrm{OH}}^{-}⇌{\mathrm{CO}}_{3}^{2-}+H_{2}O$$

When exhaled CO_2_(g) depletes CO_2_(aq) in blood, carbonic acid (H_2_CO_3_) is consumed, which triggers the equilibrium of Equation (23) to restore the level of carbonic acid by reacting bicarbonate with a hydrogen ion, exemplifying Le Châtelier's principle. This process results in making the blood more alkaline (raising pH). Similarly, when the pH is too high, the kidneys excrete bicarbonate into urine as urea via the urea cycle (or Krebs–Henseleit ornithine cycle). By removing the bicarbonate, more H^+^ is generated from carbonic acid, which comes from CO_2_(g) produced by cellular respiration.

**Figure 7:**
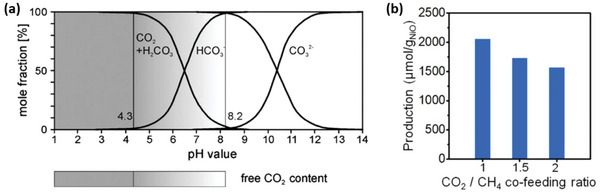
a) pH dependence of the carbonate system. Reproduced with permission.^[^ ^123^ ^]^ Copyright 2011, IOP Publishing. b) Ethanol production on a 600 nm NiO/ZnO nanorod catalyst from a CO_2_/CH_4_ feed mixture for a 2 hr reaction. Reproduced with permission.^[^ ^105^ ^]^ Copyright 2023, Elsevier.

By utilizing the HCO_3_^−^ buffer system, we can capture the produced CO_2_ as H_2_CO_3_, and convert it back to CO_3_^2−^ via HCO_3_^−^, under neutral conditions, as shown in Equations (22)–(24). A similar process can also occur under alkaline conditions via Equations (25), (26). Previous studies on CO_2_ sensors and CO_2_ reduction electrolyzers have experimentally verified the pH dependence of the HCO_3_^−^ buffer system as well.^[^ ^123^, ^124^, ^125^ ^]^ **Figure** **7**a shows that the CO_2_ level decreases to almost zero as pH increases from 4.3 to 8.2 due to the formation of HCO_3_^−^. HCO_3_^−^ dominates in the pH range of 7–10, and CO_3_^2−^ is produced from HCO_3_^−^ as pH increases from 8.2 to 14.^[^ ^87^ ^]^

Regarding the electro‐catalytic conversion of methane to alcohol employing CO_3_^2−^ as the oxidant, Moon et al. have shown that high ethanol production rates can be achieved when methane is co‐fed with CO_2_ for in situ production of CO_3_^2−^ under alkaline conditions via Equations (25), (26). However, increasing the CO_2_/CH_4_ ratio resulted in a decrease in the ethanol production rate as the pH decreases to suppress the CO_3_^2−^ formation, and the methane supply rate is low, as shown in Figure 7b.^[^ ^68^ ^]^

## 4 Challenges and Perspectives

Methane, the primary constituent of natural gas responsible for supplying ≈21% of the world's energy demand, exhibits remarkable potential as an energy source. Its combustion releases substantial energy while minimizing CO_2_ emissions. The efficient activation of methane is crucial for ensuring future energy and fuel supplies, as well as for the synthesis of valuable fine chemical feedstocks. Converting methane directly to liquid derivatives, such as methanol, via thermo‐catalytic partial oxidation, is a promising approach to utilize natural gas and reduce gas flaring. However, finding efficient, cost‐effective, and durable catalysts remains a challenge. Despite decades of research, controlling the reaction conditions to improve alcohol selectivity and yield is still a significant challenge due to the inevitable complete oxidation of methane to CO_2_ under harsh conditions. Therefore, electro‐catalysis has been proposed as an alternative to overcome the inherent limitations of thermo‐catalytic systems. Electrocatalytic methane oxidation offers several advantages, such as high energy efficiency and partial oxidation product selectivity, mainly due to operating under mild conditions.

Designing a stable and efficient electrocatalyst for methane‐to‐alcohol conversion presents several challenges. Critical factors include the selection of catalyst materials prioritizing high catalytic activity and selectivity, excellent stability, and resistance to deactivation. We highlight several key challenges that must be addressed to achieve breakthroughs in the field of electrochemical methane oxidation. First, there is a lack of mechanistic understanding of possible reaction pathways at a molecular level, primarily due to the limited number of studies dedicated to partial electrochemical oxidation of methane compared to the extensively studied complete oxidation of methane. To overcome this limitation, in situ spectro‐electrochemical studies combined with computational modeling should be conducted, enabling a deeper understanding of the underlying mechanisms.

Second, it is crucial to achieve electrolytic methane conversion at industrially relevant current densities, specifically exceeding 300 mA cm^−2^, as this is a necessary condition for practical industrial applications. Therefore, the establishment of rigorous benchmarking protocols is essential for the detection and quantification of oxidation products. Such protocols will facilitate faster development of this research frontier and its transition toward practical implementation. Departing from the traditional H‐type cell configuration, the adoption of liquid/gas‐phase electrolyzers, including liquid electrolyzer gas diffusion electrodes and membrane assembly electrodes, can contribute to addressing this challenge effectively.

Furthermore, while high‐temperature methane electro‐oxidation using oxygen‐ion conducting electrolytes has been extensively investigated, it is important to direct research efforts toward proton‐conducting electrolytes. Proton‐conducting electrolytes offer the advantage of operating at lower temperatures compared to their solid oxide counterparts. Exploring and optimizing the performance of proton‐conducting electrolytes in methane electro‐oxidation processes can broaden the range of operating conditions and enhance the feasibility of industrial‐scale applications. By addressing these challenges, we can advance the understanding, efficiency, and practicality of electrochemical methane oxidation, paving the way for its broader adoption in sustainable energy and chemical production sectors.

Lastly, the choice of oxidant generates active oxygen species on the catalyst surface to activate stable C−H bonds. Furthermore, optimizing operating conditions such as temperature, pressure, applied potential, and reactant concentrations is crucial as they have a significant impact on catalytic activity and stability. By carefully fine‐tuning these parameters, we can maximize catalytic activity while minimizing the formation of unwanted byproducts resulting from over‐oxidation. Recent developments discussed in this review address these concerns. Despite advancements in catalyst design and reactor engineering, the high thermodynamic stability of methane poses a fundamental limitation, making selective oxidation difficult and competing with OER, limiting high faradaic efficiency and selectivity. To overcome this challenge, we propose, from a theoretical perspective, using CO_3_^2−^ as the oxidant to improve conversion efficiency.

CO_3_^2−^ offer a promising alternative to conventional oxidants, such as water and oxygen gas, for electrocatalytic partial methane oxidation, due to their ability to address limitations associated with water‐based electrochemical oxidation. The mechanistic requirements for high methanol selectivity are as follows: first, O* must be weakly bound (i.e., fairly unstable) on the catalyst surface to activate methane and subsequently produce methanol. Second, a high O* coverage is desired to prevent the formation of CH_3_O* and CH_3_*, which lead to the production of other undesirable products like formaldehyde, carbon monoxide, and carbon dioxide. Water oxidant fails to meet these requirements as it is challenging to attain high coverage of fairly unstable O* on methane oxidation catalysts via electrochemical water oxidation without promoting OER. This limitation represents a fundamental obstacle in using water as the oxidant for electro‐catalytic methane‐to‐methanol conversion. In contrast, CO_3_^2−^ can efficiently generate high O* coverage at relatively low oxidation potential, thus avoiding complete oxidation via OER. Electrochemical adsorption of CO_3_^2−^ to CO_3_* (reaction occurs via a two‐electron transfer) and thermochemical dissociation into O* and CO_2_(g) becomes exothermic at low oxidation potential, offering a solution to the persistent issue when water was used as the oxidant. Using carbonate ions as the oxidant for electro‐catalytic methane conversion to alcohol raises concerns about significant CO_2_ emissions from carbonate ions. Nevertheless, there is a possibility to convert CO_2_ generated from the CO_3_^2−^ decomposition back to CO_3_^2−^ in a high pH environment, resulting in a carbon‐neutral cycle.

This review summarizes the mechanistic insights into methane activation and the design principles for catalysts based on descriptors. It also explores the impact of different oxidants and operating conditions on electrochemical partial methane oxidation, highlighting the limitations of using water‐based oxidants for this process. Our theoretical perspective suggests that the use of CO_3_^2−^ ions as the oxidant can lead to a higher chance of developing promising electro‐catalysts for methane‐to‐alcohol conversion. Carbonate ion‐based electrocatalysis is expected to continue generating interest among academics and researchers in this field. The recent advances in electrocatalysis are exciting, and future technologies for the electrochemical oxidation of methane to alcohols using CO_3_^2−^ ions are eagerly anticipated for implementation on an industrial scale.

## Conflict of Interest

The authors declare no conflict of interest.
